# Mechanical Factors Implicated in Zirconia Implant Fracture Placed within the Anterior Region—A Systematic Review

**DOI:** 10.3390/dj10020022

**Published:** 2022-02-02

**Authors:** Lauryn Attard, Victoria Lee, Jennifer Le, Chloe Lowe, Vipra Singh, Jacky Zhao, Dileep Sharma

**Affiliations:** 1 College of Medicine and Dentistry, James Cook University, Cairns, QLD 4878, Australia; lauryn.attard@my.jcu.edu.au (L.A.); victoria.lee4@my.jcu.edu.au (V.L.); jennifer.le@my.jcu.edu.au (J.L.); wyatt.lowe@my.jcu.edu.au (C.L.); vipra.singh@my.jcu.edu.au (V.S.); jacky.zhao@my.jcu.edu.au (J.Z.); 2 Australian Institute of Tropical Health & Medicine, Smithfield, QLD 4870, Australia

**Keywords:** aesthetic zone, zirconia, dental implant, metal-free

## Abstract

Background: To analyze the fracture resistance of zirconia implants within the anterior region and evaluate whether zirconia–zirconia implants can be a viable alternative to titanium implants. Methods: Four online databases (Cochrane Library, Ovid, PubMed, and Scopus) were searched for the period of January 2011 to July 2021. All studies that analyzed the in vivo clinical outcome of two-piece implants in the anterior region in English language were included. Results: The search strategy identified 242 studies. Of these studies, three studies were included for qualitative synthesis based on the pre-determined eligibility criteria. The results showed that there is significant difference in biological results, fractal behavior and other complications between one-piece and two-piece zirconia implants. Two-piece zirconia implants demonstrated favorable longevity and success rates within anterior maxillary in short-term trials. Conclusions: Although factors involved in fractures have been identified—sandblasting, implant diameter, occlusal load, age and implant coating—there is limited quantitative assessment to gauge the fracture resistance of two-piece zirconia implants. Hence, further research with long-term clinical evidence is required.

## 1. Introduction

Dental implants are increasingly being considered and established as a reliable option for the replacement of missing teeth, and titanium is widely accepted as the gold-standard material used in implant-borne reconstructions [1,2,3]. Titanium implants can pose significant aesthetic concerns in the presence of thin gingival biotypes in the anterior maxillary region, as greyish discoloration of the peri-implant tissue may become clinically noticeable due to the underlying metallic hue of titanium [2]. Moreover, recent studies have outlined renewed concerns regarding possible allergic reactions and titanium toxicity, and their role in implant failure [2,4]. Titanium and titanium alloy particles have been reported to leach into surrounding tissues, due to corrosion and wear of metallic implants [4]. As such, peri-implant bone loss may result from inflammatory reactions triggered by hypersensitivity to titanium, allergic reactions and titanium implant corrosion, leading to osseointegration failure of the dental implant [4].

Survival rates of titanium implants have been extensively researched over the past few decades with reports suggesting 67–100% survival specifically in patients with head and neck cancer after surgical treatment [5,6,7,8]. In a recent retrospective multivariable analysis, survival and overall success of titanium implants were reported to be at 88.4% and 81.3% [8]. The factors that were identified as independent risk factors for implant failure included male gender, narrow diameter, shorter length and need for bone augmentation surgery during or prior to implant placement [8].

Ceramic materials are commonly used in dentistry for various applications, such as the fabrication of crowns and bridges, orthodontic brackets and implant abutments, due to its tooth colored, excellent esthetics and high biocompatibility [9,10]. In fact, preferential use of zirconia material in full-coverage restoration of teeth to achieve optimal gingival architecture, termed ”biologically oriented tooth-preparation technique”, has been well documented [10,11]. In addition, zirconia may have reduced plaque accumulation and inflammation compared to titanium, making it a preferred alternative material for dental implants [3]. Consequently, due to the aesthetic appearance of the zirconia material, its excellent biocompatibility and established osseointegrative capabilities, placement of zirconia implants in areas of aesthetic concern have been steadily increasing.

Zirconia implant systems have been available commercially as either one-piece or two-piece implant systems for more than a decade. From a mechanical perspective, one-piece designs may be superior to the two-piece systems, due to increased fracture resistance and reduced susceptibility for low-grade temperature degradation [1]. In addition, the absence of a microgap between the assembled implant and abutment reduces the potential of hosting bacteria, thus preventing marginal inflammation and bone resorption [1,3]. However, from a practitioner’s perspective, there are multiple surgical and prosthodontic shortcomings associated with a one-piece system, as the transmucosal part of the implant cannot be detached. As such, submerged implant healing is hardly possible, and there are also limited routes to compensate for positional challenges with implants with the provisional and final restoration [1]. In cases of positional challenges, wherein in situ grinding of the zirconia abutment becomes unavoidable, fracture resistance of the implant and osseointegration are impacted due to the frictional heat produced, or zirconia particles can be release into surrounding tissue [1].

Several two-piece zirconia implants are being commercially marketed and clinically utilized wherein implant-abutment ”attachment” is usually achieved through cementing the abutment and the crown to the endosseous implant, or by mechanical means (screw). Although cement can seal the microgaps and initial adjustment of implant angulation is possible, the processes are mostly irreversible, and therefore, limited options for replacement of restorations over the implant exist. In contrast, ceramic implants restored by screw retention are still utilizing titanium screws, and hence are not truly ”metal-free”.

Previous in vitro studies have noted that the biomechanical stability of both one-piece and two-piece zirconia implants could withstand a loading duration of 10 million cycles at 95 N force [12,13]. Although the bending moment to fracture (BMF) was unaffected by loading conditions, the BMF of zirconia implants (one-piece and two-piece) were significantly less than that of the control group [12]. Furthermore, Spies et al. investigated the long term stability of a commercially available two-piece zirconia implant under the same chewing simulator used in Reimer’s study [12,14]. Results revealed that the BMF of the two-piece zirconia implant was comparable to titanium implant, but significantly less than a titanium alloy implant [12,14]. As such, Spies et al. were in agreement with Riemer’s findings that two-piece zirconia implants are capable of withstanding masticatory forces for several years [12,14].

Nonetheless, there are still concerns regarding the fracture resistance of zirconia, as standardized testing protocols addressing the aging behavior of zirconia implants have mostly focused on zirconia abutments, rather than the implant itself. Despite the fracture resistance of zirconia implants in in vitro studies being widely reported, there is a distinct lack of data derived from in vivo specifically human studies. Furthermore, Garcia-Hammaker et al. highlighted that the variability of in vitro study designs makes it difficult to have a consensus on the fracture strength and to correlate it to clinical circumstances [15]. As such, limitations on zirconia’s intended use in the clinical environment still exist, due to a lack of long-term clinical evidence of two-piece zirconia implants, as the technology has only been recently developed. Furthermore, the use of tooth-colored zirconia implants has been more prevalent in the anterior region of dentition, due to the aesthetic need of the patients. Therefore, the rationale for this systematic review was to clinically evaluate the factors influencing the fracture resistance of zirconia implants within the anterior region.

## 2. Methods

This systematic review aimed to address the following focused research question in PICOS format “What are the mechanical factors influencing fracture resistance (outcome) in two-piece (Intervention) versus one-piece zirconia implants (comparison) in humans (Population) within the anterior region?” and secondary questions included “What is the fractal behavior of anterior zirconia implants?” and “How does the longevity of two-piece zirconia implants compare to one-piece designs?”

This systematic review used the Preferred Reporting Items for Systematic Review and Meta Analyses (PRISMA) guidelines [16]. The protocol for this systematic review is registered with Prospero ID: CRD42021271790 and available on https://www.crd.york.ac.uk/prospero/display_record.php?ID=CRD42021271790 (accessed on 25 January 2022).

Studies were included if they fulfilled these criteria: all original research articles and case series, and all study designs including prospective, retrospective and randomized controlled clinical trials on patients treated with one- or two-piece zirconia implants within the anterior region of the oral cavity that qualitatively addressed the incidence and factors implicated in the fracture of zirconia implants within the anterior region. Furthermore, two-piece implants, anterior/aesthetics/aesthetic zone, zirconia and coated zirconia fracture were considered as inclusion criteria. Exclusion criteria were: implants placed in animals, in vitro studies, posterior implants, implants not fabricated with pure zirconia titanium screw/abutment/implant, lithium disilicate, Emax, porcelain-fused-to-metal, studies that were not peer reviewed, grey literature, non-English literature, case reports and reviews of any type, including systematic reviews.

A comprehensive literature search was performed using the following electronic databases: Scopus, PubMed, Ovid, and Cochrane Library in July 2021. Articles published from 1 January 2011 up to and including 16 July 2021 were considered. No other restrictions were placed during each initial search of the databases. The articles were retrieved using different combinations of MeSH terms (Appendix A).

Independently, four reviewers (VL, CL, VS, JZ) commenced the identification of relevant articles employing the predetermined search strategy, and citations of relevant articles were exported into reference management software (EndNote X8). After removal of duplicates, the screening phase was completed by the reviewers, wherein the titles and abstracts of all remaining studies were screened for studies that potentially met the inclusion criteria. In the eligibility phase, the full-text versions of all remaining studies were independently assessed by the same reviewers for eligibility for the included phase. Any disagreements over the eligibility of studies were resolved through discussion between the reviewers and arbitration by the lead of the review team (DS), to avoid inter-reviewer variability.

## 3. Results

**Figure 1:**
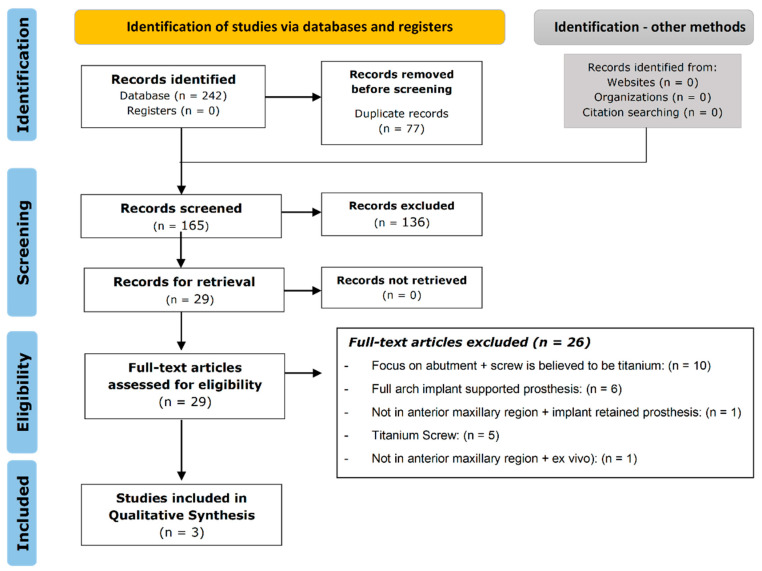
Flow diagram describing the literature screening and selection process based on the Preferred Reporting Items for Systematic Reviews and Meta-Analyses (PRISMA) guidelines [16].

The PRISMA flow chart (Figure 1) demonstrates the selection process employed for the systematic review. Study searches were completed through the following databases: Scopus, Ovid, PubMed and Cochrane. These yielded 242 articles in total. After removing all 77 duplicates, 165 studies remained with titles and abstracts that could potentially reflect the review question. Following title and abstract screening, 29 articles continued to the eligibility stage, of which a total of three studies were deemed to fulfill all the inclusion criteria. 

Relevant data were extracted and collated into the JBI SUMARI online tool by six authors independently (LA, JL, VL, CL, VS, JZ), using the data-extraction template provided [17,18]. Extracted data included country, setting/context, participation characteristics, group description, outcome measured and description of main results. Studies designated to be included for this review were arrived at after agreement from all the reviewers, and any disagreements were resolved by further discussion with the expert reviewer (DS). Furthermore, the data extracted from the included papers were assessed for accuracy by two reviewers (LA, JL) and confirmed by the expert reviewer (DS).

### 3.1. Quality Assessment of Included Studies

Full-text articles were collated and data were extracted using the JBI SUMARI Tool [19]. Six reviewers (LA, JL, VL, CL, VS, JZ) independently assessed the quality and risk of bias for each of the included studies (Table 1 and Table 2). Disagreements were resolved by further discussion and moderation by an expert reviewer (DS). Questionnaires provided by JBI SUMARI were applied and reviewed by two independent assessors for each article (Table 3 and Table 4).

### 3.2. Study Characteristics

The eligible articles were all in vivo or ex vivo studies, with one study categorized as a Randomized Control Trial (Paolantoni, et al.) [20] whilst the remaining two were both Cohort studies (Roehling and Scherrer, et al.) [21,22]. Classifying the included articles based on their study designs allowed for adequate analysis of the fracture resistance in one- and two-piece zirconia implants placed within the anterior region.

### 3.3. Risk of Bias Assessment

The quality and risk of bias assessments for the three articles included in the review were carried out using the appraisal tool created by JBI SUMARI [19,23], and findings are presented in Table 1 and Table 2. The study by Roehling et al. [21] was deemed to have low bias, while articles from Scherrer et al. [15,22] and Paolantoni et al. [20] were found to have a high level of bias. The level of bias for each article was determined by the JBI SUMARI appraisal tool [23]. Appendix B lists the JBI SUMARI Appraisal Tool questions [23].

**Table 1:** Quality Assessment and Appraisal of Included Studies—Cohort Study.

Citation	Q1	Q2	Q3	Q4	Q5	Q6	Q7	Q8	Q9	Q10	Q11
Roehling et al. (2016) [21]	Y	Y	Y	N	N/A	Y	N	Y	Y	N/A	Y
Scherrer et al. (2019) [22]	N/A	N/A	Y	N/A	N/A	N/A	Y	N/A	N/A	N/A	N/A
Percentage (%)	50	50	100	0	0	50	50	50	50	0	50

**Table 2:** Quality Assessment and Appraisal of Included Studies: Randomized Controlled Trial.

Citation	Q1	Q2	Q3	Q4	Q5	Q6	Q7	Q8	Q9	Q10	Q11	Q12	Q13
Paolantoni et al. (2016) [20]	U	N	Y	U	U	U	N	Y	Y	N/A	N/A	Y	U
Percentage (%)	0	0	100	0	0	0	0	100	100	0	0	100	0

The relevant data collected for qualitative synthesis are summarized in two custom-made characteristic tables: Randomized Clinical Trial (Table 3) and Cohort Studies (Table 4). Meta-analyses were not performed, due to heterogeneity between studies involving different samples, methodology, study models and outcomes measure.

**Table 3:** Characteristics of Included Studies—Randomized Controlled Trial.

Study	Country	Setting/Context	Participant Characteristics	Groups	Outcomes Measured	Description of Main Results
Paolantoni et al. 2016 [20]	Italy	University of Naples	Patients—65 Females—44 Males—21 74 missing maxillary teeth Mean age 53 ± 4 years.	Treatment group 1 received standard zirconia anchorage with a layer of lithium disilicate (pressed) and an all-ceramic luted crown (two piece). Group 1—Single implant-51 patients; Two single nonadjacent implants prosthetic restorations—9 patients 51 fixtures (68.9%) were placed with a one-stage procedure with a healing period of 12 months; 23 fixtures (31.1%) were inserted with a two-stage procedure and a healing period of 6 months. Group 2 (*n* = 45) received one-piece restoration with the porcelain facing fire-pressed onto custom zirconia anchorage.	Mechanical outcomes: Fracture failure of abutment, restoration and porcelain facing, loss of retention of the abutment due to screw loosening, or restoration fracture. Biological outcomes: Implant Mobility, Plaque Index (PI), Bleeding Index (BI), and marginal bone loss (MBL).	Only 2 (out of 45) one-piece restorations fractured. Screw loosening was not reported. None of the implants showed mobility. No significant variations between groups in PI, BI, and MBL at follow-up examination.

**Table 4:** Characteristics of Included Studies—Cohort Studies.

Study	Country	Setting/Context	Participant Characteristics	Groups	Outcomes Measured	Description of Main Results
Scherrer et al. 2019 [22]	Switzerland	Study design: Retrospective Cohort Study. Funding source: Not specified.	Broken first generation monotype zirconia implant parts, upper portion with the crown cemented.	Number of teeth: 15. Axis Biodental Implants (10), Z-Systems Implants (3), Straumann Implants (1), Swiss Dental Solutions Implants (1).	Details of Intervention: Fractographic failure analysis to identify origin of failure and characteristics of surface cracks. Measured/Treatment Outcomes: A mathematical model spreadsheet was utilized to compute bending and torsion moments on a total load of 500 N distributed over identified occlusal contacts. Follow-up Period: Not specified.	Transgranular propogation of fracture was noted near the origin. Addition of 0.25 wt% alumina to a 3Y-TZP can increase transgranular fracture due to increased grain–boundary cohesion. Direct relation of fracture origin to large grit alumina sandblasting (Z-System) and porous coating (AXIS Biodental) was evident. Generally, fractures initated from the periphery of the smaller diameter between two threads at the bone level. Occlusal loading to the implant’s central axis can effect bending moments and onset of fracture.
Roehling. et al. 2016 [21]	Germany	Study design: Retrospective Funding Source: Not specified.	85 participants (47 female, 38 male) who received a first generation monotype zirconia implant between the dates of Oct 2004 and Nov 2009. Mean age 54.86; 161 implants assessed, 7 smokers (11 implants), 21 patients had bruxism (57 implants).	Only 1 group measured at initial placement and at/near 7 years.	Details of intervention: Data collected-number, diameter, length and position of implants, age, gender, risk factors, and bone quality and intra-oral image. Outcome measured: Subjective complaints, recurrent peri-implantitis with suppuration, implant mobility, gingival Index (GI), modified plaque index (PI) probing depth (PD), modified sulcus bleeding Index (mBI), distance from the implant shoulder to the mucosal margin (DIM). Success was measured by a criteria formulated by Buser and colleagues. Follow-up period: Mean follow-up period of 5.94 +/− 0.09 years	125 implants survived; 36 implants lost early. Mean values noted: GI-0.03; PI-0.23; mBI-0.59; PD-2.8 mm. Radiographically mean crestal bone loss was 0.97 ± 0.07 mm and diameter-reduced implants showed lower survival rate (3.25 mm = 58.5% survival, 4 mm = 89.9%, 5 mm = 78.6%). Satisfaction with esthetical outcome of zirconia implant after 7 years-90%. 18 of the 36 failed implants were due to fracture at the sandblasted portion of the coronal part of the implant. Highest survival rate was noted for implant placement at 40–59 years of age.

## 4. Discussion

Complications with dental implants leading to structural failure whilst functional within the oral cavity for a period of time is well documented [24,25,26,27]. This is true in the case of both titanium and newer implants, such as Zirconia implants [28,29]. The clinical evidence investigating the fracture resistance of one- and two-piece zirconia anchorages used in single-tooth maxillary anterior implant-supported crowns was limited, with only one randomized clinical trial and two retrospective cohort studies identified in the database searches (Figure 1). In the randomized control trial conducted by Paolantani et al., the clinical results including incidence of complications of one-piece and two-piece implants were investigated [20]. They reported that no statistically significant difference exists in biological results, fractal behavior and other complications between one-piece and two-piece implants [20]. Furthermore, Paolantani reported the radiographically evaluated mean edgeal bone loss to be 1.17 ± 0.89 mm [20]. This biological parameter of two-piece zirconia implants was comparably investigated in another study reporting a similar value, 0.097 ± 0.07 mm for edgeal bone loss [21]. Despite the randomized control trial that reported three abutment fractures, no anchorage screw loosening occurred, and no implants were lost early or exhibited significant clinical mobility. In contrast, 36 implants (22.4%) were lost early over the previous seven-year clinical trial, with 18 of the 36 implants failing due to fracture at the threads of the coronal aspect of the implant [20].

Analyzing the two cohort studies identified a range of factors that have the greatest impact on fractal behavior and longevity in one- and two-piece zirconia implants in the anterior region: diameter, sandblasting, occlusal load, age, coating, bruxism and location. Roehling et al. reported that zirconia implants with a reduced diameter of 3.25 mm had a survival rate of 58.5% in comparison to an implant with a diameter of 4.0 mm, which had a survival rate of 88.9% [21]. In addition, Scherrer noted that the Z-lock system, manufactured to a diameter of 2.7 mm, had a poor survival rate reported at only 59.6% [22]. However, this did not imply that the higher diameter ensures greater success, as Roehling et al. reported that a 5.0 mm diameter had a success rate of 78.6%, less than an implant body diameter of 4 mm (88.9%) [21]. This may be due to the fact that when the diameter of a zirconia implant body is increased, the shear amount of stress dissipated to the implant shoulder region is reduced, increasing the fractal resistance of the implant and therefore its survivability [22]. Both studies agreed that the diameter-reduced implants of less than 3.6 mm were not suitable for clinical use [22]. Consequently, a modified design of 3.6 mm has now been introduced, with its application limited to the anterior incisor region of both maxillary and mandibular dentition [22].

Surface characteristics, both macro and micro features incorporated into the implant surface, have been known to influence the osseointegration and thereby the survival of dental implants. Two studies discussed the impact of using alumina large-grit sandblasting on one- and two-piece zirconia implants [21,22]. They noted that fractures often occurred at the sandblasted section of the implant body due to the creation of surface defects, hindering long-term survivability [22]. However, sandblasting is known to have a positive effect, as it contributes to phase-transformation toughening, changing zirconia from a tetragonal phase to a monoclinic phase, which results in volume expansion, compression of cracks and increased fracture resistance [22].

The magnitude, frequency, duration and direction of the occlusal load endured on the implant-supported tooth has been well recognized as a factor in the overall success of implant therapy. When an implant undergoes occlusal loading under eccentric force, the force is dissipated to the supporting bone tissue. As such, the implant body must be designed in such a way as to reduce micro-movements and correct force dissipation. Scherrer et al. noted that the fractures of the first-generation implants developed under the cyclic load of masticatory forces [22]. Furthermore, the combination of smaller implant diameter (2.7 mm), and sandblasted (large grit) surface treatment was noted to be unfavorable for the survival of the implant, since the fracture origins were associated with the sandblasting indentations [22]. With respect to specific loading sites, the mesio-buccal cusps of crowns were noted as ”prime contacts” that received up to 3/4 of the 500 N load applied, to create a fracture. Furthermore, 75% of anterior zirconia implants failed palato-buccally, due to contact-loading forces on the palatal surface of the maxillary central incisor crown. An exception to this was the Z-Look 3 implants, which fractured bucco-palatally during silicone impression retrieval [21]. Interestingly, this study also reported that nine implants were lost from four patients due to fractures caused by bruxism, however five of these were noted in one patient [21].

The relation between patient age and implant survival was reported in one of three included studies. Roehling et al. noted that the highest seven-year survival rate was in patients between 40–59 years, compared to patient groups aged between 19–39 years or 60–85 years [21]. This highlights that there could be a relation between age and successful implant therapy. However, the results from Roehling et al. account for implant failure that has fractured or undergone incomplete osseointegration [21]. Therefore, more research is needed to establish correlation between patient age and implant success.

Fractographic analysis of the implant microstructure has been used to confirm the presence of a transgranular fracture within the bulk of the implant, suggesting that this occurs when cracks or defects are inherent within the coating and arise during processing prior to sintering of the implants. These cracks present within the coating were reported to reach the implant bulk and influenced the location of crack propagation [22]. Furthermore, preexisting large cracks within the coating are not eliminated in final implant processing, with in vitro testing also suggesting a substantial reduction (18%) in strength, compared to uncoated implants [22]. Although the potential consequences of failure during the coating process is evident, the information surrounding its impact on fracture resistance and implant survival is unknown and requires further research [22].

All included studies acknowledged the limitations within their study design. Due to the inherent limitations of a retrospective cohort study, the authors were unable to perform follow-up or comparative analysis of their results, thus making it difficult to relate to clinical-based scenarios due to limited information on the original scenario for implant fracture. Specifically, the Roehling et al. study on zirconia implant fracture, although insightful, came with its limitations [21]. Assessor bias, patient information and selection and implant system were the factors identified that had the capacity to influence data on zirconia fracture. Scherrer et al. noted significant challenges in differentiating between occlusal contacts with wear evidence and occlusal adjustments on crowns, as some roughness from polishing overlaid localized wear from contacts [22]. Furthermore, a highly unusual fracture site was recorded in an implant retrieved from a patient with bruxism—mid-way along the implant inside the bone. The authors deemed this result inconclusive and attributed this to a lack of participants exhibiting similar sites of fracture. Scherrer et al. also noted the limitations of biting force (500 N) employed, as the load responsible for the fracture of each implant in the study was unknown and hence the values presented in this study may not be accurate [22].

Although the study by Paolantani et al. is a randomized control trial, the risk of selection bias is high, as the assignment of participants to treatment groups were chosen with coin-toss randomization [20]. This creates issues with concealment, validation and reproducibility, whereas simple randomization with less risk of bias could have been achieved through a computer-generated sequence. Moreover, performance bias could have influenced the survivability rate of implants in the Paolantani et al. study, as treatment groups were not identically treated, and guided bone regeneration was required for 26 implants [20]. As such, the inconsistencies in patient selection criteria between both studies may influence the overall survival rates. Nonetheless, despite limited long-term clinical results for two-piece zirconia implants, the data gathered by Paolantani et al. seem to demonstrate that single zirconia anchorages have favorable short-term mechanical and biological outcomes within the anterior maxillary region [20].

Evolution in ceramic manufacturing technology has led to significant and predictable improvements in the characteristic of dental materials. Recently, the focus has shifted towards additive manufacturing techniques for fabrication of customized root-form zirconia implants utilizing a combination of stereolithography and digital light processing [15,30]. However, further in vitro and clinical research is essential to confirm its suitability for root-form dental implant manufacture.

There were some limitations within this systematic review. Only three studies satisfied all the inclusion criteria limiting the extent of data available for consideration. Furthermore, this paper only considered implants placed in anterior dentition, and the factors that were implicated in this review may not be applicable to posterior dentition due to a significant difference in the magnitude and direction of forces applied on the implant.

## 5. Conclusions

This systematic review discusses the application of zirconia implant designs within the anterior region and their associated factors influencing fracture and incidence. Despite short term trials, literature confirms the benefits of anterior zirconia implants having good technical and biological results, and there is currently limited clinical evidence on the long-term results of two-piece zirconia implants. Although various factors implicated in zirconia implant fractures have been identified as sandblasting, implant diameter, occlusal load, age and implant coating, there is limited quantitative assessment to gauge the fracture resistance of two-piece zirconia implants clinically. Hence, further research into long-term survival rates to provide evidence on the fracture resistance of two-piece zirconia implants is required, to support evidence-based designs and guidelines for routine clinical use, specifically in the anterior region of the oral cavity.

## Data Availability

No new data were created or analyzed in this study. Data sharing is not applicable to this article.

## Appendix A

**Table A1:** Databases searched, and MeSH terms used.

Database	MeSH Terms and Search Strategy
Scopus	(“fract* resistan*” OR “fract* analys*” OR “fract* behavio*r” OR “fract* strength” OR fract* OR “fract* dimension” OR “fract* tough*” OR “fract* model” OR fragment* OR break* OR crack* OR fissure* OR split*) AND (“two-piece zirconia” OR “two piece zirconia” OR “2 piece zirconia” OR “2-piece zirconia” OR “zirconia implant*” OR “zirconia screw” OR “zirconia abutment*”) AND TITLE-ABS-KEY (anterior OR “anterior area” OR “anterior zone” OR “anterior section” OR “anterior region” OR “anterior segment” OR maxil* OR “*esthetic zone” OR “*esthetic region” OR “*esthetic area” OR “*esthetic section” OR “*esthetic segment”) AND TITLE-ABS-KEY (implant* OR abut*))
Ovid	fract* resistan*.mp. fract* analys*.mp. fract* behavio*r.mp. fract* strength.mp. fract*.mp. fract* dimension.mp. fract* tough*.mp. fract* model.mp. fragment*.mp. break*.mp. crack*.mp. fissure*.mp. split*.mp. two-piece zirconia.mp. two piece zirconia.mp. 2 piece zirconia.mp. 2-piece zirconia.mp. zirconia implant*.mp. zirconia screw.mp. zirconia abutment*.mp. anterior.mp. anterior area.mp. anterior zone.mp. anterior section.mp. anterior region.mp. anterior segment.mp. maxil*.mp. esthetic zone.mp. aesthetic zone.mp. esthetic region.mp. aesthetic region.mp. esthetic area.mp. aesthetic area.mp. esthetic section.mp. aesthetic section.mp. esthetic segment.mp. aesthetic segment.mp. implant*.mp. abut*.mp. 1 or 2 or 3 or 4 or 5 or 6 or 7 or 8 or 9 or 10 or 11 or 12 or 13 14 or 15 or 16 or 17 or 18 or 19 or 20 21 or 22 or 23 or 24 or 25 or 26 or 27 or 28 or 29 or 30 or 31 or 32 or 33 or 34 or 35 or 36 or 37 38 or 39 40 and 41 and 42 and 43
PubMed	(maxilla OR maxil* OR anterior OR “anterior area” OR “anterior region” OR “anterior zone” OR “anterior section” OR “aesthetic zone” OR “aesthetic area” OR “esthetic zone” OR “esthetic area”) AND (fract* OR “fracture resistance” OR “fractal behaviour” OR “fracture behaviour” OR “fracture strength” OR “fractal dimension” OR “fracture toughness” OR “fracture model” OR “fractal model” OR break* OR crack* OR fissure* OR split*) AND (“two piece zirconia” OR “two-piece zirconia” OR “2-piece zirconia” OR “2 piece zirconia” OR “zirconia abutment” OR “zirconia implant” OR zirconia implants”)
Cochrane Library	(“fract* resistan*” OR “fract* analysis” OR fracture OR fractures OR “fract* behaviour” OR “fracture strength” OR “fract* dimension” OR “fractal toughness” OR “fracture toughness” OR “fractal model” OR “fracture model” OR fragment* OR break* OR crack* OR fissure* OR split*) AND (“two-piece zirconia” OR “two piece zirconia” OR “2 piece zirconia” OR “2-piece zirconia” OR “zirconia abutment*” OR “zirconia screw” OR “zirconia implant*”) AND (anterior OR “anterior area” OR “anterior zone” OR “anterior section” OR “anterior region” OR “anterior segment” OR maxil* OR “aesthetic zone” OR “esthetic zone” OR “aesthetic region” OR “*esthetic region” OR “aesthetic area” OR “*esthetic area” OR “aesthetic section” OR “*esthetic section” OR “aesthetic segment” OR “*esthetic segment”)

## Appendix B

**Table A2:** Jbi Sumari Appraisal Tools [23].

**A. JBI Critical Appraisal Checklist for Randomized Controlled Trials [23].**					
	**Criteria**	**Yes**	**No**	**Unclear**	**Not Applicable**
1	Was true randomization used for assignment of participants to treatment groups?				
2	Was allocation to treatment groups concealed?				
3	Were treatment groups similar at the baseline?				
4	Were participants blind to treatment assignment?				
5	Were those delivering treatment blind to treatment assignment?				
6	Were outcomes assessors blind to treatment assignment?				
7	Were treatment groups treated identically other than the intervention of interest?				
8	Was follow-up complete and if not, were differences between groups in terms of their follow-up adequately described and analyzed?				
9	Were participants analyzed in the groups to which they were randomized?				
10	Were outcomes measured in the same way for treatment groups?				
11	Were outcomes measured in a reliable way?				
12	Was appropriate statistical analysis used?				
13	Was the trial design appropriate, and any deviations from the standard RCT design (individual randomization, parallel groups) accounted for in the conduct and analysis of the trial?				
	Overall appraisal: Include/Exclude/Seek further info				
**B. JBI Critical Appraisal Checklist for Cohort Studies [23].**					
	**Criteria**	**Yes**	**No**	**Unclear**	**Not Applicable**
1	Were the two groups similar and recruited from the same population?				
2	Were the exposures measured similarly to assign people to both exposed and unexposed groups?				
3	Was the exposure measured in a valid and reliable way?				
4	Were confounding factors identified?				
5	Were strategies to deal with confounding factors stated?				
6	Were the groups/participants free of the outcome at the start of the study (or at the moment of exposure)?				
7	Were the outcomes measured in a valid and reliable way?				
8	Was the follow-up time reported and sufficient to be long enough for outcomes to occur?				
9	Was follow-up complete, and if not, were the reasons for lack of follow-up described and explored?				
10	Were strategies to address incomplete follow-up utilized?				
11	Was appropriate statistical analysis used?				
	Overall appraisal: Include/Exclude/Seek further info				

## References

[1] Bethke A., Pieralli S., Kohal R.J., Burkhardt F., von Stein-Lausnitz M., Vach K., Spies B.C. (2020). Fracture resistance of zirconia oral implants in vitro: A systematic review and meta-analysis. Materials.

[2] Kunrath M.F., Gupta S., Lorusso F., Scarano A., Noumbissi S. (2021). Oral tissue interactions and cellular response to zirconia implant-prosthetic components: A critical review. Materials.

[3] Comisso I., Arias-Herrera S., Gupta S. (2021). Zirconium dioxide implants as an alternative to titanium: A systematic review. J. Clin. Exp. Dent..

[4] Kim K.T., Eo M.Y., Nguyen T.T.H., Kim S.M. (2019). General review of titanium toxicity. Int. J. Implant Dent..

[5] Katsoulis J., Fierz J., Iizuka T., Mericske-Stern R. (2013). Prosthetic rehabilitation, implant survival and quality of life 2 to 5 years after resection of oral tumors. Clin. Implant. Dent. Relat. Res..

[6] Javed F., Al-Hezaimi K., Al-Rasheed A., Almas K., Romanos G.E. (2010). Implant survival rate after oral cancer therapy: A review. Oral Oncol..

[7] Hessling S.A., Wehrhan F., Schmitt C.M., Weber M., Schlittenbauer T., Scheer M. (2015). Implant-based rehabilitation in oncology patients can be performed with high long-term success. J. Oral Maxillofac. Surg..

[8] Hasegawa T., Sasaki A., Saito I., Arimoto S., Yatagai N., Hiraoka Y., Takeda D., Kakei Y., Akashi M. (2021). Success of dental implants in patients with large bone defect and analysis of risk factors for implant failure: A non-randomized retrospective cohort study. Clin. Oral Investig..

[9] Munro T., Miller C.M., Antunes E., Sharma D. (2020). Interactions of osteoprogenitor cells with a novel zirconia implant surface. J. Funct. Biomater..

[10] Hadyaoui D., Daouahi N., Nouira Z., Cherif M. (2014). Gingival harmony in anterior aesthetic restorations. Dent. J..

[11] Agustin-Panadero R., Serra-Pastor B., Fons-Font A., Sola-Ruiz M.F. (2018). Prospective clinical study of zirconia full-coverage restorations on teeth prepared with biologically oriented preparation technique on gingival health: Results after two-year follow-up. Oper. Dent..

[12] Kohal R.-J., Dennison D.K. (2020). Clinical longevity of zirconia implants with the focus on biomechanical and biological outcome. Curr. Oral Health Rep..

[13] Riemer L.K.R. (2018). Ceramic implant systems and the influence of artificial hydrothermal aging on their breaking strength—An in vitro study. Dent. Prosthet. Clin..

[14] Spies B.C., Fross A., Adolfsson E., Bagegni A., Doerken S., Kohal R.J. (2018). Stability and aging resistance of a zirconia oral implant using a carbon fiber-reinforced screw for implant-abutment connection. Dent. Mater..

[15] Garcia-Hammaker S., Saglik B., Sierraalta M., Razzoog M. (2021). Influence of screw channel angulation on the fracture resistance of zirconia abutments: An in vitro study. J. Prosthodont..

[16] Page M.J., McKenzie J.E., Bossuyt P.M., Boutron I., Hoffmann T.C., Mulrow C.D., Shamseer L., Tetzlaff J.M., Akl E.A., Brennan S.E. (2021). The PRISMA 2020 statement: An updated guideline for reporting systematic reviews. PLoS Med..

[17] Moola S., Munn Z., Tufanaru C., Aromataris E., Sears K., Sfetcu R., Currie M., Qureshi R., Mattis P., Lisy K., Aromataris E., Munn Z. (2017). Systematic reviews of etiology and risk. Joanna Briggs Institute Reviewer’s Manual.

[18] Tufanaru C., Munn Z., Aromataris E., Campbell J., Hopp L., Aromataris E., Munn Z. (2017). Systematic reviews of effectiveness. Joanna Briggs Institute Reviewer’s Manual.

[19] J.B. Institute (2019). Joanna Briggs Institute (JBI) SUMARI. https://sumari.jbi.global/.

[20] Paolantoni G., Marenzi G., Blasi A., Mignogna J., Sammartino G. (2016). Findings of a four-year randomized controlled clinical trial comparing two-piece and one-piece zirconia abutments supporting single prosthetic restorations in maxillary anterior region. Biomed. Res. Int..

[21] Roehling S., Woelfler H., Hicklin S., Kniha H., Gahlert M. (2016). A Retrospective clinical study with regard to survival and success rates of zirconia implants up to and after 7 years of loading. Clin. Implant. Dent. Relat. Res..

[22] Scherrer S.S., Mekki M., Crottaz C., Gahlert M., Romelli E., Marger L., Durual S., Vittecoq E. (2019). Translational research on clinically failed zirconia implants. Dent. Mater..

[23] Joanna Briggs Institute (2018). Critical Appraisal Tools.

[24] Liaw K., Delfini R.H., Abrahams J.J. (2015). Dental implant complications. Semin. Ultrasound. CT MRI.

[25] Dutta S.R., Passi D., Singh P., Atri M., Mohan S., Sharma A. (2020). Risks and complications associated with dental implant failure: Critical update. Natl. J. Maxillofac. Surg..

[26] Do T.A., Le H.S., Shen Y.W., Huang H.L., Fuh L.J. (2020). Risk factors related to late failure of dental implant—A systematic review of recent studies. Int. J. Environ. Res. Public Health.

[27] Castellanos-Cosano L., Rodriguez-Perez A., Spinato S., Wainwright M., Machuca-Portillo G., Serrera-Figallo M.A., Torres-Lagares D. (2019). Descriptive retrospective study analyzing relevant factors related to dental implant failure. Med. Oral Patol. Oral Cir. Bucal.

[28] Bosshardt D.D., Chappuis V., Buser D. (2017). Osseointegration of titanium, titanium alloy and zirconia dental implants: Current knowledge and open questions. Periodontol. 2000.

[29] Afrashtehfar K.I., del Fabbro M. (2020). Clinical performance of zirconia implants: A meta-review. J. Prosthet. Dent..

[30] Osman R.B., van der Veen A.J., Huiberts D., Wismeijer D., Alharbi N. (2017). 3D-printing zirconia implants; a dream or a reality? An in-vitro study evaluating the dimensional accuracy, surface topography and mechanical properties of printed zirconia implant and discs. J. Mech. Behav. Biomed. Mater..

